# Association between perfluoroalkyl substances exposure and thyroid function in adults: A meta-analysis

**DOI:** 10.1371/journal.pone.0197244

**Published:** 2018-05-10

**Authors:** Min Joo Kim, Shinje Moon, Byung-Chul Oh, Dawoon Jung, Kyunghee Ji, Kyungho Choi, Young Joo Park

**Affiliations:** 1 Department of Internal Medicine, Seoul National University College of Medicine, Seoul, Republic of Korea; 2 Division of Endocrinology and Metabolism, Department of Internal Medicine, Hallym University College of Medicine, Seoul, Republic of Korea; 3 Department of Physiology, Lee Gil Ya Cancer and Diabetes Institute, Gachon University College of Medicine, Incheon, Republic of Korea; 4 Division of Environmental Health, Korea Environment Institute, Sejong, Republic of Korea; 5 Department of Occupational and Environmental Health, Yongin University, Yongin, Republic of Korea; 6 School of Public Health, Seoul National University, Seoul, Republic of Korea; Universidade do Porto Faculdade de Medicina, PORTUGAL

## Abstract

**Objective:**

Many people are exposed to perfluoroalkyl substances (PFASs) because these substances are widely used as industrial products. Although epidemiological studies suggest that PFASs can disrupt thyroid hormones, the association between PFAS exposure and thyroid function remains inconclusive. Therefore, we performed a comprehensive meta-analysis to investigate the association between PFASs exposure and thyroid hormones.

**Methods:**

We searched medical literature databases for articles on the association between PFASs–perfluorooctanesulfonic acid (PFOS), perfluorooctanoic acid (PFOA), and perfluorohexane sulfonic acid (PFHxS)–and thyroid hormone levels in adults. Twelve articles were included in the meta-analysis, and the pooled z values were calculated with correlation or regression coefficients.

**Results:**

The blood PFOS concentration was positively correlated with free T4. The pooled z value was 0.05 (95% confidence interval (CI): 0.03, 0.08). PFOS was negatively correlated with total T4 and total T3 when excluding outlier studies. In a subgroup analysis stratified by mean PFOS concentration, PFOS was observed to be positively associated with free T4 and TSH and negatively associated with total T3 in the intermediate concentration group (8–16 ng/mL). PFOA concentration was negatively correlated with total T4 (z value, -0.06; 95% CI: -0.09, -0.03) after omitting one outlier study. PFHxS also showed a negative correlation with total T4 (z value, -0.04; 95% CI: -0.07, -0.01). A subgroup analysis of pregnant women showed that there was no association between PFASs and thyroid hormones.

**Conclusions:**

Our meta-analysis suggests that PFASs are negatively associated with total T4, and their effect can be different depending on the PFAS concentration.

## Introduction

Perfluoroalkyl substances (PFASs; or previously described as perfluorinated compounds) have been widely used as various industrial products such as surfactants, lubricants, photographic emulsifiers, paints, fire-fighting foams and food packaging. Humans are exposed to PFASs mainly through contaminated food, water, and household dust [1]. Thus, PFASs are detected in >95% of the general population [2]. There has been concern about potential adverse effects of PFASs on human health because animal studies have indicated that PFASs could cause tumors and neonatal deaths [3, 4]. Moreover, human studies have reported that exposure to perfluorooctanesulfonic acid (PFOS) and perfluorooctanoic acid (PFOA), types of PFASs, are associated with decreased birth weight and fertility [5, 6]. Therefore, PFOS and perfluorooctane sulfonyl fluoride were added to Annex B of the Stockholm Convention on Persistent Organic Pollutants in 2009. Consequently, PFASs have been regulated in many countries, including the USA [7]. Regulation has resulted in a decreased serum concentration of PFASs in recent studies conducted in the USA, Australia, and Asia [8–11]. However, PFASs are persistent in the environment because of their high stability and long half-lives in humans [12, 13] and are still widely used in some countries, including China [14]. Therefore, PFASs are still detected in most people, and it has been reported that blood concentrations of some less restrictive PFASs like perfluorohexane sulfonic acid (PFHxS) and perfluorononanoic acid (PFNA) have remained unchanged or have increased in Sweden and Japan [15–17]. Thus, PFASs remain some of the important environmental pollutants that can lead to serious health problems [5, 6].

Thyroid hormones play a critical role in the regulation of metabolism, and thyroid function is related to cardiovascular disease, fertility, and fetal neurodevelopment [18, 19]. In animal experiments, treatment of PFAS induced hypertrophy or hyperplasia of thyroid follicular cells in rat [20] and lowered total and free T4 concentrations [21, 22]. It has been suggested that some PFASs may disrupt the thyroid hormone system in humans. Because PFOS, PFOA, and PFHxS are the most widely present PFASs, their association with thyroid dysfunction has been most studied than other PFASs [23, 24]. However, the association is still inconclusive. Blood PFAS concentrations are negatively correlated with thyroid hormone concentrations according to some studies [23, 25, 26], while other studies showed positive correlations [27, 28] or no association [29–32]. These inconsistent results may be due to the concentration-dependent differential effects of PFASs. It is generally believed that chemicals have monotonic linear dose-response curves; thus, a high-dose of a chemical is more toxic. This concept has been changing particularly with respect to the adverse effects of endocrine-disrupting chemicals. Some studies indicate that endocrine-disrupting chemicals might have nonmonotonic or U-shape dose responses; thus, a lower-dose of chemicals could be more harmful [33, 34]. Therefore, we conducted a meta-analysis to investigate whether blood PFAS concentrations were correlated with thyroid hormone levels, particularly pertaining to different PFAS concentrations in adults.

## Materials and methods

A meta-analysis was performed in accordance with the general principles recommended in the Preferred Reporting Items for Systematic Reviews and Meta-Analyses (PRISMA) (S1 Appendix) [35].

### Search strategy

In the databases PubMed, Embase, and Web of Science, articles, published from inception (1985 in PubMed and Web of Science and 1987 in Embase) to April 30, 2017 were searched by two investigators (S.M. and M.J.K.) using a combination of the following terms: “perfluorinated”, “perfluorooctanoic”, “perfluorooctane”, “perfluorohexane”, “PFOS”, “PFOA”, and “PFHxS” and “thyroid”. The language used in the literature was limited to English. The detailed search strategy is presented in the S2 Appendix.

### Eligibility criteria

To select studies to be included in this meta-analysis, the PCOS (participants, interventions, comparators, outcomes, and study design) framework was used [36]. The participants of interest were adults (aged ≥ 18 years) from the general population. Children and infants were excluded in this analysis. Studies that measured exposures to PFOS, PFOA, and/or PFHxS and thyroid hormone levels such as total/free thyroxine (T4), total triiodothyronine (T3), and thyroid-stimulating hormone (TSH) in blood were included. Studies that presented thyroid status as categorized groups such as hyperthyroidism or hypothyroidism, were excluded. Outcomes of interest were the association between PFASs and thyroid hormone levels. Articles that gave the Pearson correlation coefficient, Spearman correlation coefficient, or regression coefficient were included. Cross-sectional, case-control, and cohort studies were included.

### Study selection

**Fig 1:**
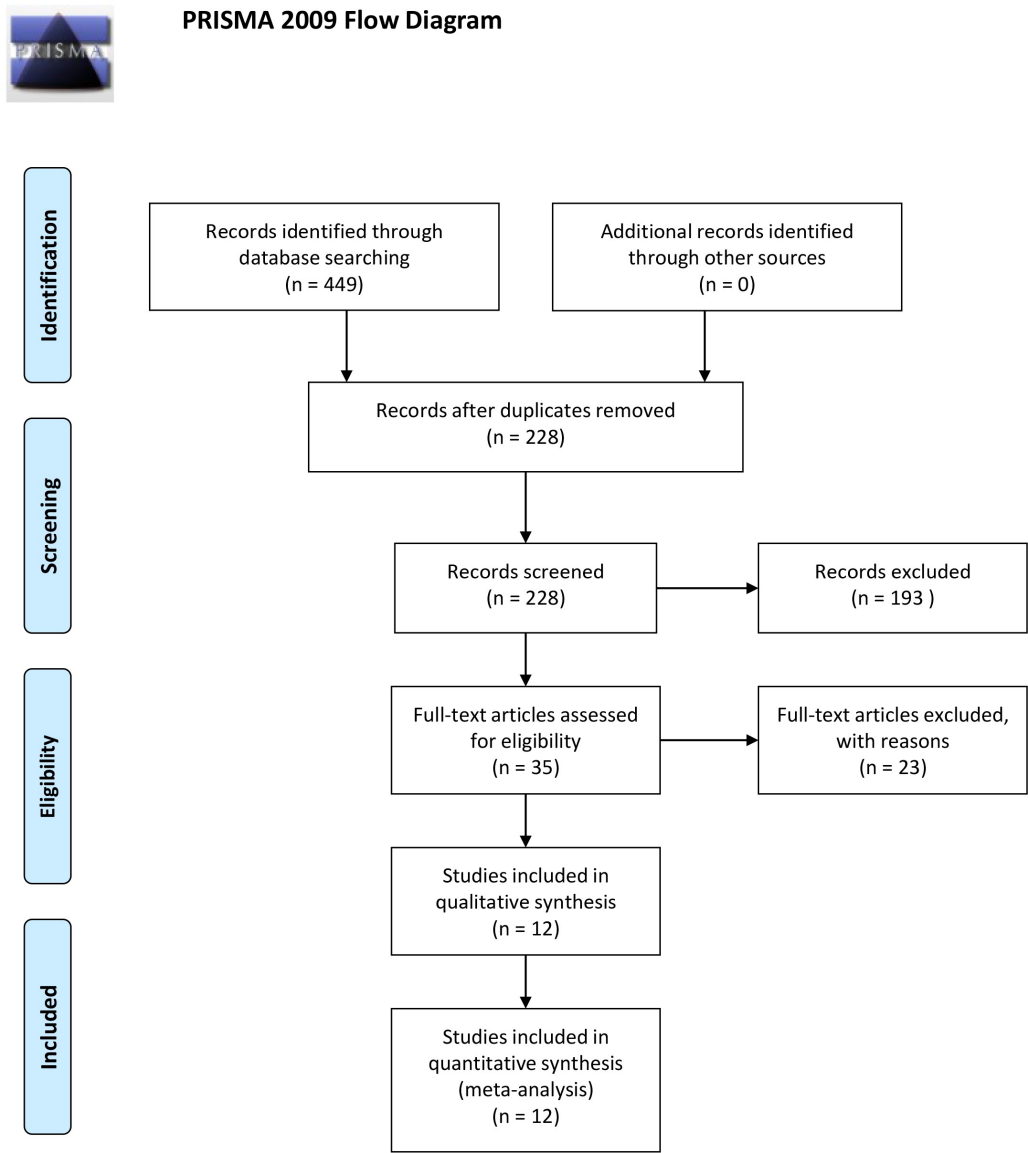
Representation of the search strategy based on PRISMA flow diagram.

Literature search yielded 449 potentially relevant articles (Fig 1). Duplicate articles were excluded, and the latest or most relevant article was included if the studies had multiple reports. Thus, 228 articles were screened, and the studies were selected by a two-step method. First, titles and abstracts were screened according to the eligibility criteria. Articles were excluded for the following reasons: 1) the study was published in abstract form, as an expert opinion, as a letter, as a conference article, or as a review (n = 57); 2) the study used animals or *in vitro* models (n = 105); 3), the study was not related to PFAS and thyroid (n = 32). Second, the full texts of the selected, potentially relevant articles (n = 35) were electronically downloaded and reviewed independently by the two investigators (S.M. and M.J.K.) based on the criteria listed above. Articles were excluded for the following reasons: 1) the participants were not adults (n = 8) or part of the general population (n = 2); 2) PFAS and/or thyroid concentrations were presented as quartiles or quintiles, not as a continuous variable (n = 9); 3) there was no information on the Pearson correlation coefficient, Spearman correlation coefficient, or regression coefficient between PFASs and thyroid hormones (n = 2); 4) multiple studies used the National Health and Nutrition Examination Survey database (n = 2). Any disagreements were resolved by a third investigator (Y.J.P.). Finally, 12 articles were selected for the meta-analysis.

### Data collection

The following data were collected: first author, publication year, country, number of subjects, the mean or median blood concentration of PFASs, TSH, total/free T4 and/or total T3, and Pearson correlation coefficient, Spearman correlation coefficient, or regression coefficient. In addition, the method for measuring free T4 in each study was checked. Blood concentration of free T4 was measured in nine studies and all of them used the analog method: chemiluminescent immunoassay (n = 5), radioimmunoassay (n = 3), or enzyme-linked immunosorbent assay (n = 1).

### Assessment of bias risk

Three researchers independently assessed the methodological quality of the included articles using a modified cross-sectional assessment provided by the Agency for Healthcare Research and Quality [37]. Here, eight items were used to assess the quality, and all articles scored in the six to eight range (S1 Table). We concluded that the quality of these cross-sectional studies did not affect the quality of our meta-analysis.

### Data analyses and statistical methods

To investigate the association between PFASs and thyroid hormone levels, we calculated the pooled z values using a Pearson correlation coefficient transformed by the Fisher z-transformation. Among the 12 studies, the Pearson correlation coefficient was reported only in two studies [28, 38]. In three studies [23, 31, 39], we conducted a re-analysis of the available raw data to determine the Pearson correlation coefficients. In other studies, the Pearson correlation coefficient was calculated from the existing Spearman correlation coefficient or regression coefficient with a corresponding 95% confidence interval (CI) using the following formulas [40–42]:

(1) Estimated Pearson correlation coefficient = 2 × sin (Spearman correlation coefficient ×π/6)
(2) (Estimated Pearson correlation coefficient)^2^ = t^2^/(t^2^+n-2)

t = regression coefficient /the standard error of regression coefficient.

The Higgins’ I^2^ statistic was used to test for heterogeneity. Subgroup and sensitivity analyses were performed to determine the cause of heterogeneity. In the sensitivity analysis, we checked the changes in the results by excluding one specific study to examine the stability or strength of the results. The potential for publication bias was assessed using a funnel plot analysis. All statistical analyses were conducted using the statistical program R (R version 3.1.0, 2014, www.r-project.org).

## Results

### Characteristics of eligible studies

Four hundred and forty-nine studies were screened and assessed for eligibility, and 12 studies were finally selected for this meta-analysis (Fig 1). The study characteristics are summarized in Table 1. All considered studies were cross-sectional studies, and the sample size of each study ranged from 31 to 1,950 subjects. The mean age (29–64 years) and sex ratio (0%–100%) of each study varied.

**Table 1:** Characteristics of studies included.

Study	Sampling Year	Location	Population	N	Age (years) Mean	Sex Number	Chemical (ng/mL) Geometric mean (95% CI) or median (range)				Thyroid hormone	Statistical analysis
							PFOS	PFOA	PFHxS	Others		
Bloom et al. 2010	1995–1997	USA	Sportfish anglers and their partners	31	39	M:27 F:4	19.57 (16.30–23.50)	1.33 (1.15–1.53)	0.75 (0.52–1.06)	PFDA PFNA PFUnDA	TSH Free T4	Multiple linear regression models (Log transformed)
Crawford et al. 2017	2008–2009	USA	Women attempting to conceive	99	33.3	All F	9.29 (8.31–10.38)	2.79 (2.48–3.16)	1.59 (1.37–1.84)	PFNA	TSH Total T4 Free T4 Total T3	Pearson correlations
Dallaire et al. 2009	2004	Canada	General population (Inuit)	506	36.8	M: 245 F: 378	18.28 (17.19–19.44)				TSH Free T4 Total T3	Multiple linear regression models (Log transformed)
Ji et al. 2012	2008	Korea	General population	556	42.5	M: 219 F: 337	7.96^a^ (5.58–12.10)^b^	2.74^a^ (2.04–3.64) ^b^	1.51^a^ (0.92–2.34) ^b^	PFHpS PFNA PFDA PFUnDA PFDoDA PFTrDA	TSH Total T4	Multiple linear regression models (Log transformed)
Kato et al. 2016	2001–2005	Japan	Pregnant women (24–41 weeks of gestational age)	392	31.1	All F	5.2^a^ (1.6–12.3)	1.2^a^ (LOD–3.4)			TSH Free T4	Spearman correlations
Lewis et al. 2015	2011–2012	USA	General population (NHANES)	1682	40	M: 858 F: 824	10	2.55	1.85	PFNA	TSH Total T4 Free T4 Total T3 Free T3	Multiple linear regression models
Raymer et al. 2012	2002–2005	USA	IVF Clinic	246	41.6	All M	32.3^a^ (6.4–151.0)^c^	9.2^a^ (1.3–66.3)^c^			TSH Total T4 Total T3	Multiple linear regression models
Shrestha et al. 2015	2005, 2010	USA	General population (Riverside)	87	63.6	M: 51 F: 36	31.60 (5.29–139.53)^c^	9.17 (0.58–42.69)^c^			TSH Total T4, Free T4 Total T3	Pearson correlations (Log transformed)
Wang et al. 2013	1999–2008	Norway	Pregnant women (17–18 weeks of gestational age)	903	30	All F	12.77 (12.45–13.10)	2.13 (2.07–2.20)	0.62 (0.59–0.64)	PFDA PFHpS PFNA PFUnDA	TSH	Multiple linear regression models (Log transformed)
Wang et al. 2014	2000–2001	Taiwan	Pregnant women	283	28.8	All F	12.73^a^ (9.65–17.48)^b^	2.39^a^ (1.54–3.40) ^b^	0.81^a^ (0.30–1.35) ^b^	PFNA PFDeA PFUnDA PFDoDA	TSH Total T4, Free T4 Total T3	Multiple linear regression models (Log transformed)
Wen et al. 2013	2007–2010	USA	General population (NHANES)	1181	NA	M: 672 F: 509	14.2 (13.59–14.86)	4.15 (4.02–4.29)	2.00 (1.89–2.11)	PFNA	TSH Total T4, Free T4 Total T3, Free T3	Multiple linear regression models (Log transformed)
Yang et al. 2016	2013	China	Pregnant women	157	29.8	All F	4.23 (0.73–19.96)^c^	1.74 (0.73–8.11)^c^	0.53 (0.12–4.22)^c^	PFNA PFDA PFUnA PFDoA	TSH Total T4 Free T4 Total T3 Free T3	Spearman correlations

CI, Confidence interval; M, Male; F, Female

^a^ Median

^b^ Interquartile range

^c^ Range (Min–Max)

### The correlation between PFOS exposure and thyroid function

**Fig 2:**
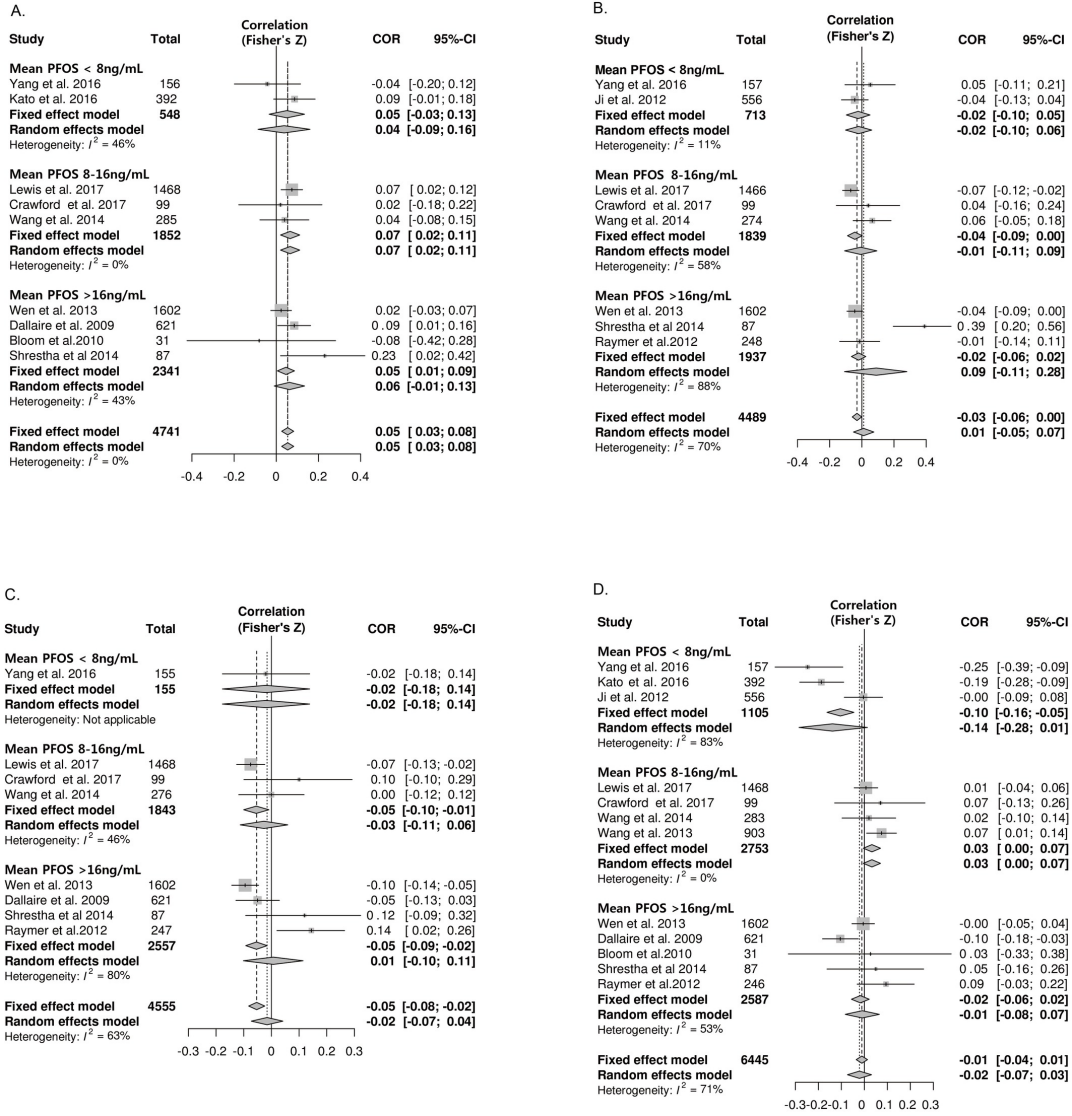
Forest plots of the summary z value with corresponding 95% CIs for the correlation between PFOS and thyroid hormone. **A.** Correlation between PFOS and free T4. **B.** Correlation between PFOS and total T4. **C**. Correlation between PFOS and total T3 **D.** Correlation between PFOS and TSH.CI, confidence interval; W, weight.

Nine studies provided data suitable for a meta-analysis of correlations between PFOS exposure and free T4 (Fig 2A). PFOS was positively correlated with free T4 and the pooled z value was 0.05 (95% CI: 0.03, 0.08) without any significant heterogeneity (I^2^ = 0%). No publication bias was found. To evaluate the concentration-dependent difference in the association, studies were divided into three groups according to their mean PFOS concentration: < 8 ng/mL (low), 8–16 ng/mL (intermediate), and > 16 ng/mL (high). The correlation became more pronounced in the intermediate concentration group; the pooled z values of the studies were 0.07 (95% CI: 0.02, 0.11; I^2^ = 0%). Because pregnancy itself can affect thyroid hormone levels and the thyroid function plays an important role in fetal neurodevelopment [43, 44], the association between PFASs and thyroid hormones in pregnant women was analyzed separately (Table 2). However, no significant correlation between PFOS and free T4 was observed in the subgroup of pregnant women, while a significant correlation was found in the subgroup with the general population (z value 0.06; 95% CI: 0.02, 0.09; I^2^ = 18%).

**Table 2:** Association between PFAS and thyroid hormone according to the pregnancy status.

	Pregnant women			General population		
	No of studies	Pooled z value	I^2^	No of studies	Pooled z value	I^2^
**PFOS**						
**Free T4**	3	0.05 (-0.02; 0.11)	0	6	0.06 (0.02; 0.09)	18
**Total T4**	2	0.06 (-0.03; 0.15)	0	6	0.00 (-0.07; 0.07)	74
**Total T3**	2	-0.01 (-0.10; 0.09	0	6	-0.01 (-0.08; 0.06)	72
**TSH**	4	-0.08 (-0.12; 0.08)	89	8	-0.01 (-0.04; 0.02)	27
**PFOA**						
**Free T4**	3	0.00 (-0.07; 0.06)	0	5	0.01 (-0.02; 0.05)	11
**Total T4**	2	0.04 (-0.06; 0.13)	0	6	-0.03 (-0.09; 0.04)	69
**Total T3**	2	0.04 (-0.05; 0.14)	29	5	0.05 (-0.01; 0.11)	56
**TSH**	4	0.00 (-0.05; 0.04)	44	6	0.00 (-0.03; 0.04)	32
**PFHxS**						
**Free T4**	2	0.01 (-0.01; 0.05)	0	4	0.02 (-0.01; 0.05)	0
**Total T4**	2	0.01 (-0.18; 0.20)	74	4	-0.04 (-0.07; -0.01)	1
**Total T3**	2	-0.01 (-0.16; 0.14)	57	3	0.01 (-0.03; 0.04)	0
**TSH**	3	0.00 (-0.12; 0.13)	74	5	0.00 (-0.04; 0.03)	0

Eight studies were suitable for meta-analysis of correlations between PFOS exposure and total T4. Unlike free T4, the pooled z value between PFOS and total T4 was insignificant (z value 0.01; 95% CI: -0.05, 0.07) and showed significant heterogeneity (I^2^ = 70%; p < 0.01) (Fig 2B). One outlier study was found using sensitivity analysis [28]. After omitting that study, PFOS was found to be negatively correlated with total T4 (z value -0.04; 95% CI: -0.07, -0.01; I^2^ = 5%) (S2 Table). Subgroup analyses based on the mean PFOS concentration and pregnancy status showed no correlation (Fig 2B and Table 2).

Eight studies were included for a meta-analysis of correlations between PFOS and total T3. PFOS was not associated with total T3 (z value -0.02; 95% CI: -0.07, 0.04) and showed significant heterogeneity (I^2^ = 63%) (Fig 2C). One outlier study was found using sensitivity analysis [30]. After omitting that study, PFOS was found to be negatively correlated with total T3 (z value -0.04; 95% CI: -0.06, -0.01) (S2 Table). In the subgroup analysis based on the mean PFOS concentration, PFOS also showed a negative correlation with total T3 in the intermediate concentration group (z value -0.05; 95% CI: -0.10, -0.01; I^2^ = 46%). A subgroup analysis based on pregnancy status showed no correlation (Table 2).

Twelve studies were used for meta-analysis of correlations between PFOS exposure and TSH. The pooled z value between PFOS and TSH was -0.02 (95% CI: -0.07, 0.03) with considerable heterogeneity (I^2^ = 71%) (Fig 2D). Even after omitting two outlier studies [25, 26] found by the sensitivity analysis, no significant correlation was observed (z value 0.01; 95% CI: -0.02, 0.03). In the subgroup analysis stratified by mean PFOS concentration, a significant positive correlation between PFOS and TSH in the intermediate group was found (z value 0.03; 95% CI 0.00, 0.07) (Fig 2D). PFOS was not associated with TSH in pregnant women or the general population (Table 2).

### The correlation between PFOA exposure and thyroid function

**Fig 3:**
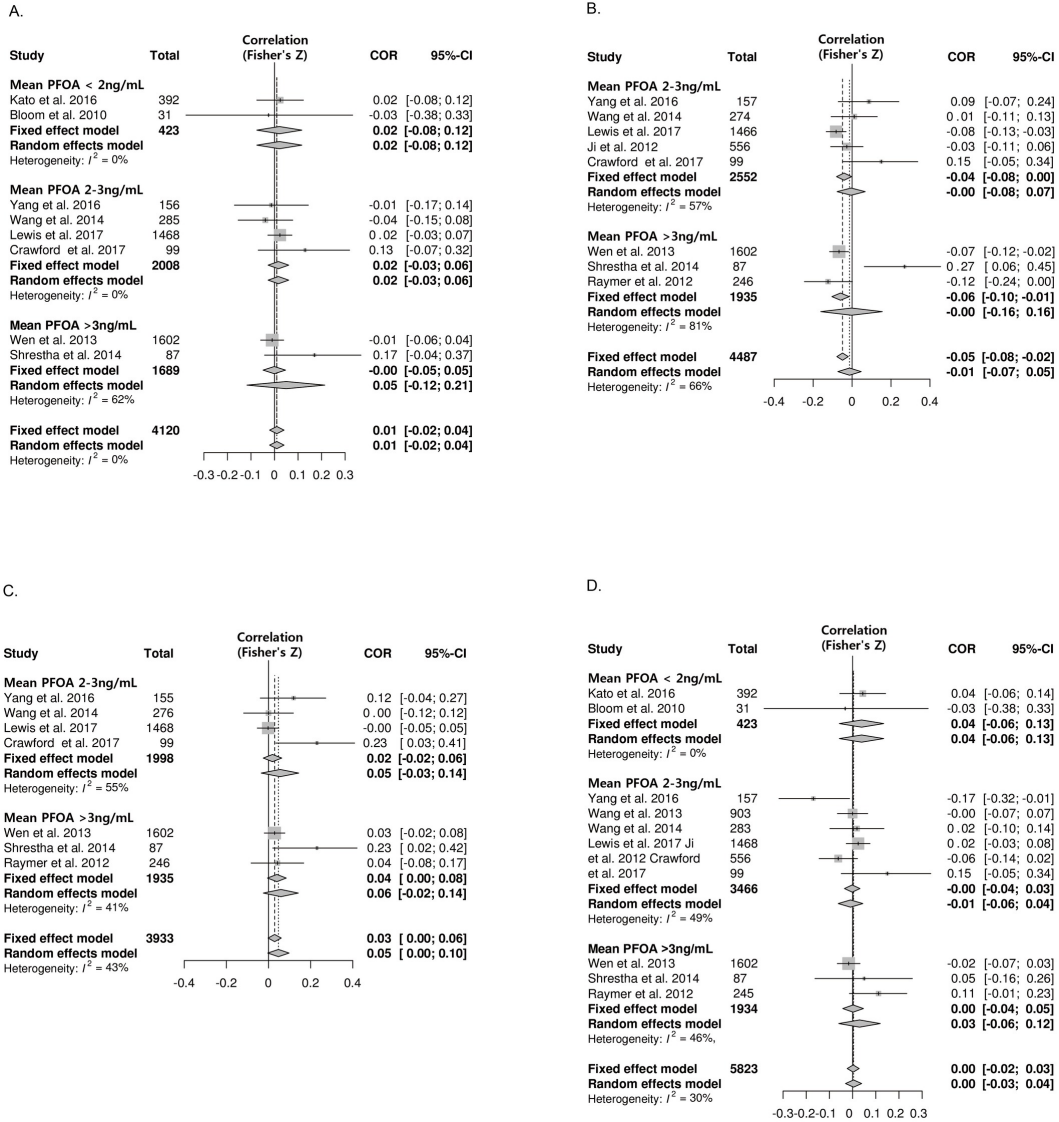
Forest plots of the summary z value with corresponding 95% CIs for the correlation between PFOA and thyroid hormone. **A.** Correlation between PFOA and free T4. **B.** Correlation between PFOA and total T4. **C**. Correlation between PFOA and total T3 **D.** Correlation between PFOA and TSH.CI, confidence interval; W, weight.

Eleven studies were included in the meta-analysis of correlations between PFOA exposure and thyroid hormones: eight for free T4, eight for total T4, seven for total T3, and eleven for TSH. There was no significant correlation between PFOA exposure and total/free T4 (Fig 3A and 3B). The pooled z value between PFOA and total T4 was -0.01 (95% CI: -0.07, 0.05) with significant heterogeneity (I^2^ = 66%). One outlier study was found using sensitivity analysis [28]. The study by Shrestha et al. has distinct characteristics [28]; the mean age of this study (63 years) was the highest compared with the other studies (28–41 years), and the mean PFOA level was relatively high (9.1 ng/mL) compared to the other studies. After omitting the one outlier study, the pooled z value between PFOA and total T4 showed a modest negative correlation (z value -0.06; 95% CI: -0.08, -0.03; I^2^ = 47%) (S2 Table). PFOA showed a modest correlation with total T3 (z value 0.03; 95% CI: 0.00, 0.06; I2 = 43%) (Fig 3C). There was no significant association between PFOA exposure and TSH (Fig 3D).

The studies were divided into three groups according to the mean PFOA concentration: < 2 ng/mL (low), 2–3 ng/mL (intermediate), and > 3 ng/mL (high). The subgroup analysis stratified by either the PFOA levels or pregnancy revealed no significant correlation (Fig 3 and Table 2).

### The correlation between PFHxS exposure and thyroid function

**Fig 4:**
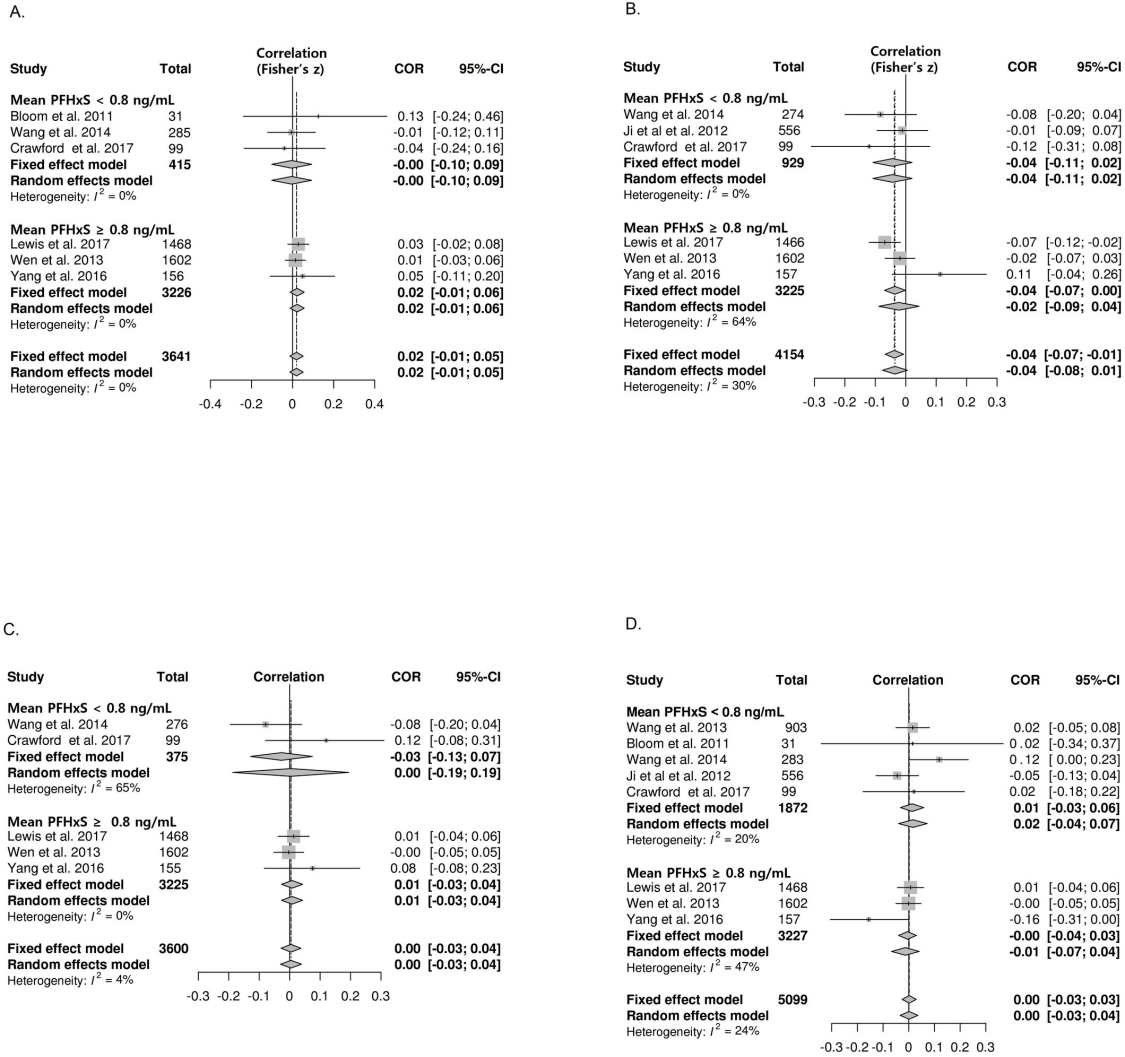
Forest plots of the summary z value with corresponding 95% CIs for the correlation between PFHxS and thyroid hormone. **A.** Correlation between PFHxS and free T4. **B.** Correlation between PFHxS and total T4. **C**. Correlation between PFHxS and total T3 **D.** Correlation between PFHxS and TSH.CI, confidence interval; W, weight.

Eight studies met the eligibility criteria for meta-analysis of correlations between PFHxS exposure and thyroid hormones: Six for free T4, six for total T4, five for total T3, and eight for TSH. There were no associations between PFHxS and free T4, total T3, or TSH (Fig 4). The studies were divided into two groups according to the mean PFHxS concentration: < 0.8 ng/mL (low), and ≥ 0.8 ng/mL (high). The subgroup analysis stratified by the mean PFHxS level revealed no correlations between PFHxS and thyroid hormone levels (Fig 4). PFHxS was negatively correlated with total T4 (z value -0.04; 95% CI -0.07, -0.01; I^2^ = 30%) (Fig 4C). Subgroup analysis based on pregnancy status showed a significant correlation in the general population (z value -0.04; 95% CI: -0.07, -0.01; I^2^ = 30%) while there was no significant correlation in pregnant women (Table 2).

## Discussion

Whether exposure to PFASs can disrupt thyroid function remains uncertain because previous epidemiological studies have reported conflicting results regarding the relationship. This is the first meta-analysis that provides evidence on the health effects of PFASs as a thyroid disrupting chemical; although a systematic review on the association between PFASs and thyroid function was reported previously [45], the subjects of that study were limited to pregnant women and their children and a meta-analysis was not performed. In this study, we performed meta-analysis of the correlation between PFASs and thyroid hormone in adults, and found PFASs and thyroid hormone levels to be associated. The strongest correlation was observed in studies with intermediate mean PFOS concentrations; PFOS was positively correlated with free T4 and TSH and negatively correlated with total T4. The results suggest that PFASs could induce thyroid dysfunction and disease. The association between PFOA exposure and thyroid disease has been reported in some studies which were not included in our meta-analysis [24, 46].

Our results suggest that PFOS exposure could increase free T4 levels and decrease total T4 levels. In all the studies included in our analysis, free T4 was measured by the analog method, which is subject to a methodological issue. PFOS can bind to thyroid hormone binding proteins, such as albumin and transthyretin (TTR) competing with thyroid hormone [47, 48], and this competition could result in an increase of circulating free thyroid hormone [49]. In rats, the change of free T4 observed after PFAS treatment, when measured by the analog method, disappeared when measured by equilibrium dialysis [50]. However, no significant difference in free T4 was observed in human serum with change in measurement method [51]. In humans, thyroxine-binding globulin (TBG) is the main thyroid hormone binding protein, whereas TTR is the main binding protein in rodents [52]. While most PFASs bind with TTR, only a few PFASs bind with TBG, and its binding affinity is low [53]. Therefore, the effects of PFASs on binding proteins may be much weaker in humans than in rodents. However, our results show that total/free T4 is inversely related to PFOS and suggest that PFOS may interfere with the binding of thyroid hormone and binding proteins in humans.

All three studied PFASs were negatively correlated with total T4 in the meta-analysis. There is sufficient evidence showing that PFASs lower total T4. Experimental studies performed in rats and monkeys have reported that PFOS or PFOA treatment lowered the total and/or free T4 concentrations [21, 22, 54, 55]. Increased hepatic degradation of thyroid hormone by PFOS-induced UDP-glucuronyltransferase (UGT) was suggested as a possible underlying mechanism [22, 49]. However, the results of animal studies may not be relevant in humans because the metabolic rates for xenobiotics in rodents are much higher than those in human [56]. Thus, the half-life of PFASs in rodents is only a few days while it takes years for humans to metabolize PFOS [12, 13, 57]. Moreover, peroxisome proliferation mechanism, which has little role in humans, has an important role in the metabolism of xenobiotics in rodents [58].

The effect of PFASs can be different according to the concentration. Therefore, we performed a subgroup analysis. Interestingly, PFOS showed a significant correlation with thyroid hormones in the intermediate concentration group (8–16 ng/mL). It was not a typical linear dose-response relationship. Such nonmonotonic or U-shaped dose response is considered to be one of the characteristics of endocrine-disrupting chemicals [33, 34].

Pregnancy can affect the influence of PFASs on thyroid function. Maternal PFASs can be transferred to fetuses, and the PFAS concentration in cord sera is positively correlated with PFASs in maternal serum [26]. Therefore, many studies have been done on pregnant women to investigate whether maternal exposure to PFASs could affect fetal outcomes, and there are reports that PFAS concentrations in cord blood are associated with lower birth weight or neurodevelopment status [59, 60]. Considering the crucial influence of thyroid function on fetal development [19], we can suppose that those adverse outcomes correlated with PFASs might be mediated, at least partially, by PFAS-induced changes in maternal thyroid hormone levels. Fetal development is influenced by maternal thyroid hormone throughout pregnancy, especially in the first half of gestation. Moreover, several studies have reported that maternal PFOS concentrations are associated with maternal thyroid hormone levels and fetal thyroid hormone levels [25, 61]. However, there are also studies that report no association between maternal PFAS concentrations and maternal or fetal thyroid hormone levels [62]. Therefore, to clarify the association in pregnant women, we performed a subgroup analysis of the relationship between PFAS exposure and maternal thyroid hormone levels for comparison with the general population. In this meta-analysis, we found no significant association between PFASs and thyroid function among pregnant women. However, a recent systematic review suggested a positive association between maternal PFAS exposure and TSH levels [45]. Our result may be affected by the timing of the PFAS exposure because each study took blood samples at different times during pregnancy.

Our study has limitations. Although we performed subgroup analyses based on the mean PFAS concentration and pregnancy status, there are potential confounding factors such as age and gender. Both PFAS and thyroid hormone levels vary with age and gender [2, 31]. However, Dallaire et al. [27] reported that the positive association between PFOS and free T4 was significant after adjustment for age and gender, and Shrestha et al. [28] reported that increasing age may potentiate the association between PFOA and total T4. In addition, it is possible that PFASs may have a greater impact on certain populations with autoimmune diseases or low iodine conditions. It was reported that the correlation between PFASs and thyroid hormone was stronger in subjects with high thyroid peroxidase (TPO) antibody levels and low iodine [63, 64]. However, we could not perform a subgroup analysis based on TPO and iodine status because of the limited number of studies that measured these factors. Further studies that include populations which high risk of thyroid dysfunction are required.

Because people are simultaneously exposed to multiple PFAS chemicals and other endocrine-disrupting chemicals, chemical co-exposures can be a potential confounding factor or have mixture effects. Although there was a study that reported results based on the sum of total PFAS concentrations, most studies evaluated the relationship between single PFAS and thyroid hormones [29]. The effects of PFAS mixtures on thyroid function may differ from those of single PFASs [65]. Further studies are required.

In conclusion, this meta-analysis suggests a negative correlation between certain PFASs and total T4 levels. Among PFASs, PFOS had the greatest effect on thyroid hormones, especially at intermediate concentrations (8–16 ng/mL).

## Supporting information

S1 Appendix PRISMA checklist. (DOC)

S2 Appendix Search strategy in each database. (DOC)

S1 Table Quality assessment of included studies. (DOC)

S2 Table Summary of associations between PFAS and thyroid hormone. (DOC)
