# Supplementary material for: Association between perfluoroalkyl substances exposure and thyroid function in adults: A meta-analysis
Source: PLoS One. 2018 May 10;13(5):e0197244. doi: 10.1371/journal.pone.0197244 (PMC5945046; doi:10.1371/journal.pone.0197244)
Supplement: S2 Appendix — (DOC) [file pone.0197244.s002.doc]

**S1 Appendix. Search strategy in each database.**

**PubMed**

**(((((((perfluorinated) OR perfluorooctanoic) OR perfluorooctane) OR perfluorohexane) OR PFOS) OR PFOA) OR PFHxS ) AND thyroid Filters: Publication date to 2017/04/30; English**

Filters: Publication date to 2017/04/30

Filters: English

Search #1 (All Fields): ((((((perfluorinated) OR perfluorooctanoic) OR perfluorooctane) OR perfluorohexane) OR PFOS) OR PFOA) OR PFHxS

→ Search result: 4791

Search #2 (All Fields): (#1) AND thyroid

→ Search result: 112

**Embase**

**((perfluorinated OR perfluorooctanoic OR perfluorooctane OR perfluorohexane OR pfos OR pfhxs) NOT [30-4-2017]/sd AND [english]/lim) AND (thyroid NOT [30-4-2017]/sd AND [english]/lim)**

Limit to : Records added to Embase: date to 2017/4/30

Limit to : English

Search #1 (All Fields): (perfluorinated OR perfluorooctanoic OR perfluorooctane OR perfluorohexane OR pfos OR pfhxs) NOT [30-4-2017]/sd AND [english]/lim

→ Search result: 5281

Search #2 (All Fields): thyroid NOT [30-4-2017]/sd AND [english]/lim

→ Search result: 199960

Search #3: #1 AND #2

→ Search result: 155

→ Excluded 11 articles which published after 30 April 2017

→ Search result: 144

**Web of Science**

Search #1: TOPIC: (perfluorinated) OR TOPIC: (perfluorooctanoic) OR TOPIC: (perfluorooctane) OR TOPIC: (perfluorohexane) OR TOPIC: (PFOS) OR TOPIC: (PFOA) OR TOPIC: (PFHxS)

Refined by: LANGUAGES: ( ENGLISH )

Timespan: 1864-2017.

→ Search result: 10956

Search #2: TOPIC: (thyroid)

Refined by: LANGUAGES: ( ENGLISH )

Timespan: 1864-2017.

→ Search result: 242003

Search #3: #1 AND #2

→ Search result: 207

→ Excluded 14 articles which published after 30 April 2017

→ Search result: 193
