# Supplementary material for: Association between perfluoroalkyl substances exposure and thyroid function in adults: A meta-analysis
Source: PLoS One. 2018 May 10;13(5):e0197244. doi: 10.1371/journal.pone.0197244 (PMC5945046; doi:10.1371/journal.pone.0197244)
Supplement: S1 Table — (DOC) [file pone.0197244.s003.doc]

**S1 table. Quality assessment of included studies.**

|  | **Define the source of information** | **List inclusion and exclusion criteria** | **Indicate time period used for identifying patients** | **Indicate whether or not subjects were consecutive if not population-based** | **Describe any assessments undertaken for quality assurance purposes** | **Explain any patient exclusions from analysis** | **Describe how confounding was assessed** | **The percentage or number of patients for which incomplete data** | **Total** |
| --- | --- | --- | --- | --- | --- | --- | --- | --- | --- |
| **Bloom et al. 2010** | 1 | 0 | 1 | 1 | 1 | 0 | 1 | 1 | 6 |
| **Crawford et al. 2017** | 1 | 1 | 1 | 1 | 1 | 0 | 1 | 1 | 7 |
| **Dallaire et al. 2009** | 1 | 1 | 1 | 1 | 1 | 1 | 1 | 1 | 8 |
| **Ji et al. 2012** | 1 | 1 | 1 | 1 | 1 | 1 | 1 | 1 | 8 |
| **Kato et al. 2016** | 1 | 1 | 1 | 1 | 0 | 1 | 0 | 1 | 6 |
| **Lewis et al. 2015** | 1 | 1 | 1 | 1 | 1 | 1 | 1 | 1 | 8 |
| **Raymer et al. 2012** | 1 | 0 | 1 | 1 | 1 | 0 | 1 | 1 | 6 |
| **Shrestha et al. 2015** | 1 | 1 | 1 | 1 | 1 | 1 | 1 | 1 | 8 |
| **Wang et al. 2013** | 1 | 0 | 1 | 1 | 1 | 1 | 1 | 1 | 7 |
| **Wang et al. 2014** | 1 | 0 | 1 | 1 | 1 | 0 | 1 | 1 | 6 |
| **Wen et al. 2013** | 1 | 1 | 1 | 1 | 1 | 1 | 1 | 1 | 8 |
| **Yang et al. 2016** | 1 | 1 | 1 | 1 | 1 | 1 | 1 | 0 | 7 |
