# Supplementary material for: Association between perfluoroalkyl substances exposure and thyroid function in adults: A meta-analysis
Source: PLoS One. 2018 May 10;13(5):e0197244. doi: 10.1371/journal.pone.0197244 (PMC5945046; doi:10.1371/journal.pone.0197244)
Supplement: S2 Table — (DOC) [file pone.0197244.s004.doc]

**S2 table. Summary of associations between PFAS and thyroid hormone,**

|  | **No of studies** | **Pooled z value** | **I^2^** | **Outlier studies** | **Corrected z value** | **I^2^** |
| --- | --- | --- | --- | --- | --- | --- |
| **PFOS** |  |  |  |  |  |  |
| **Free T4** | 9 | 0.05 (0.03; 0.08) | 0 | - | - | - |
| **Total T4** | 8 | 0.01 (-0.05; 0.07) | 70 | 1 | -0.04 (-0.07; -0.01) | 5 |
| **Total T3** | 8 | -0.02 (-0.07; 0.04) | 63 | 1 | -0.06 (-0.09; -0.03) | 31 |
| **TSH** | 12 | -0.02 (-0.07; 0.03) | 71 | 2 | 0.01 (-0.02; 0.03) | 39 |
| **PFOA** |  |  |  |  |  |  |
| **Free T4** | 8 | 0.01 (-0.02;0.04) | 0 | - | - | - |
| **Total T4** | 8 | -0.01 (-0.07; 0.05) | 66 | 1 | -0.06 (-0.08; -0.03) | 47 |
| **Total T3** | 7 | 0.03 (0.00; 0.06) | 43 | - | - | - |
| **TSH** | 11 | 0 (-0.02; 0.03) | 30 | - | - | - |
| **PFHxS** |  |  |  |  |  |  |
| **Free T4** | 6 | 0.02 (-0.01; 0.05) | 0 | - | - | - |
| **Total T4** | 6 | -0.04 (-0.07; -0.01) | 30 | - | - | - |
| **Total T3** | 5 | 0 (-0.03; 0.04) | 4 | - | - | - |
| **TSH** | 8 | 0 (-0.03;0.03) | 24 | - | - | - |
